# Editorial: Integrated omics approaches to accelerate plant improvement

**DOI:** 10.3389/fpls.2024.1397582

**Published:** 2024-03-20

**Authors:** Mohsen Yoosefzadeh Najafabadi, Lewis Lukens, Germano Costa-Neto

**Affiliations:** ^1^ Department of Plant Agriculture, University of Guelph, Guelph, ON, Canada; ^2^ Seed Analytics and Decision Science, Syngenta Seeds, Ithaca, NY, United States

**Keywords:** abiotic stresses, biotic stresses, biotechnology, omics-based research, plant growth promoting bacteria, plant growth

In the near future, the human population is estimated to reach 10 billion, causing an increased demand for food, feed, and other plant products. To meet these demands, it is crucial to accelerate the genetic improvement of key plant species. To increase the quality and quantity of plant products, advances in cultivation practices and or advances in genetics are necessary. Historically, traditional methods have concentrated on exploring the rearrangement of genetic variability through selection and in-field evaluations to enhance the value of complex traits. However, these traits are naturally influenced by numerous factors across the different levels of the central dogma of molecular biology. Therefore, integrating data from various ‘omics’ fields—including genomics, transcriptomics, and metabolomics—can significantly contribute to a deeper understanding and improvement of these complex traits. Recent advancements in the utilization of large datasets have shown promising results in accurately predicting desirable traits. These efforts involve studying various ‘omics’ levels, from molecular biology to agricultural environments. However, the scale and complexity of these datasets present challenges that necessitate novel insights and analytical methods to facilitate informed decision-making in plant breeding.

Efforts to enhance crop improvement have largely focused on trait means. Trait variability is also an important trait. A variety that has a favorable trait in a range of environments is more valuable than a variety whose trait varies depending on external stimuli. In Raffo et al., researchers used advanced breeding lines and genetic testing to investigate the heritability and correlation between genetic effects and environmental sensitivity in wheat grain yield. They found that micro-environmental sensitivity is heritable and can be reduced without affecting yield. They also developed a genomic prediction model that had good accuracy in predicting genetic effects on both grain yield and environmental sensitivity, suggesting that it could be a useful tool for breeding for stable, improved yield and resilience in wheat.

In Ma et al., a low-density genotyping platform containing 5.5K SNP markers was successfully developed for use in genetic research and molecular breeding in maize. Two populations were used to validate the platform, with results showing high polymorphic information content and genetic diversity values. This tool may help more breeders and geneticists capture genomic information from their populations. Levina et al. developed new methods for analyzing and improving economically important traits in potatoes, such as chip quality. By using network analysis, they were able to group hundreds of metabolic features into 44 modules, which were then used for genetic mapping and correlation with important traits. These modules were associated with specific genetic markers and were also correlated with chip color. Interestingly, metabolite features within a single module were often from distinct metabolic processes, suggesting that linkage disequilibrium caused unrelated compounds to have correlated abundances across potato genotypes.

Modern plant improvement efforts must account for the external variability present in the surrounding environment. A critical aspect to investigate is the detection and quantification of the plant microbiome’s significance. In this context, Ferrarezi et al. elucidated the interactions between the use of plant growth-promoting bacteria (PGPB) and chemical fertilizers, plants, and their microbiomes. This research examined the impact of PGPB inoculation on the microbial communities within the bulk soil and rhizosphere of maize and compared these effects across two different soil types. The results showed that PGPB had a weak growth-promoting effect on maize in undiluted soil but had a positive effect in diluted and irradiated soil. There were also changes in microbial diversity and abundance in response to PGPB inoculation, indicating the importance of the host-microbiome association. Furthermore, certain bacterial pathways, such as those involved in plant stress response, were found to be positively affected by PGPB. This study highlights the significance of considering the entire holobiont (host and associated microbes) in plant management and offers new insights for optimizing the use of microbial products. In a Dong et al., the focus was on understanding the regulatory mechanisms of anther development in the lily variety ‘Oriental Siberia’. Through transcriptome analysis of anthers at various developmental stages, the researchers identified genes and processes involved in anther development (**Figure 1**). Pollen release is a crucial trait for understanding a lily’s value to the consumer. This information can prevent pollen pollution and maintain the commercial value of cut flowers. Additionally, the silencing of two genes involved in hormone levels and pollen release resulted in an inhibited anther dehiscence phenotype, providing new insights into the regulation of anther development in lilies and other plants.

**Figure 1:**
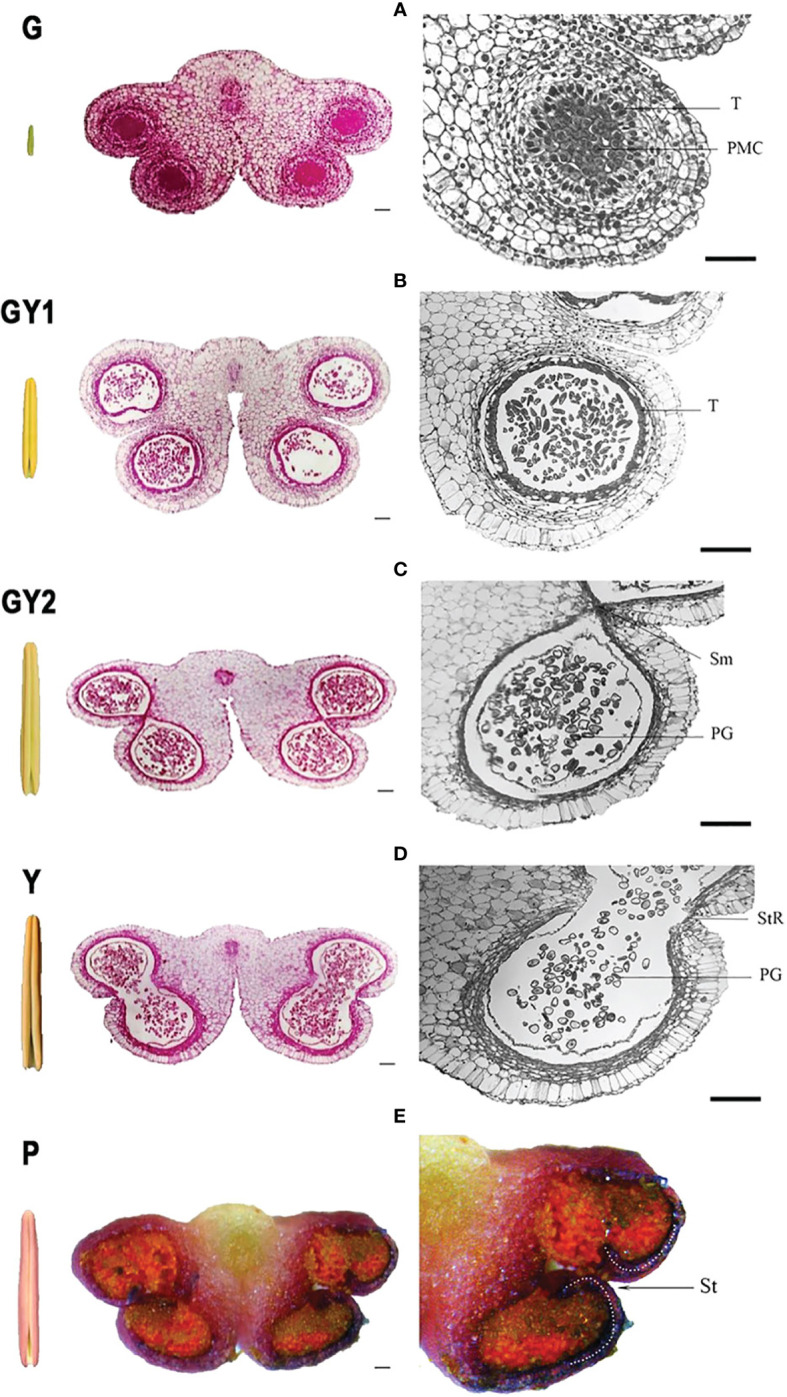
Anatomical structure of the anther at five developmental stages in Lilium Oriental Hybrid ‘Siberia’. Each stage includes a global external view of the anther, a transverse section of the complete anther with four locules, and a section of a single anther locule. **(A)** green stage (G), **(B)** green-to-yellow 1 stage (GY1), **(C)** green-to-yellow 2 stage (GY2), **(D)** yellow stage (Y), and **(E)** purple stage (P). Bar = 100 μm. Figure is taken from Dong et al..

Finally, the study of biotic stresses can reveal a complex interplay among various omics disciplines, as well as fields like physiology and plant pathology. In exploring the use of novel technologies to address biotic stresses, Kaur et al. evaluated the resistance of a diverse panel of 365 bread wheat varieties from around the world to four virulent races of Pt (leaf rust), caused by the fungus *Puccinia triticina*. The majority of the genotypes were found to be susceptible to all four races, but some genotypes had high resistance. A genome-wide association study (GWAS) analysis revealed 27 marker-trait associations for leaf rust resistance, with 20 of them being in the vicinity of previously known genes or QTLs. These findings also identified seven potentially novel genes for leaf rust resistance. Genes of interest were also identified as potential factors in disease resistance. The identified resistant lines and SNPs can serve as valuable resources for future wheat rust resistance breeding programs.

## Author contributions

MN: Writing – review & editing, Writing – original draft. LL: Writing – review & editing. GC-N: Writing – review & editing.

